# Efficacy of intravenous immunoglobulin in the treatment of recurrent spontaneous abortion: A systematic review and meta‐analysis

**DOI:** 10.1111/aji.13615

**Published:** 2022-08-30

**Authors:** Yimin Shi, Dongmei Tan, Baozhen Hao, Xiurong Zhang, Wei Geng, Yayu Wang, Jianyi Sun, Yue Zhao

**Affiliations:** ^1^ Shandong Provincial Maternal and Child Health Care Hospital Jinan City Shandong Province China

**Keywords:** intravenous immunoglobulin, meta‐analysis, obstetrics, randomised clinical trials, recurrent spontaneous abortion

## Abstract

**Objective:**

We aimed to evaluate the efficacy of IVIG in the treatment with patients with recurrent spontaneous abortion (RSA).

**Methods:**

PubMed, Embase, Web of science, Cochrane library were searched for randomized controlled (RCTs) about effect of IVIG on RSA from inception to August 20, 2021. Values of standardized mean differences (SMD) were determined for continuous outcomes.

**Results:**

A total of 15 articles involving 902 patients were included in meta‐analysis. Compared with the control group, IVIG can increase the live birth rate of recurrent spontaneous abortion patients [OR = 3.06, 95%CI (1.23, 7.64, *P* = .02]. However, recurrent abortion was divided into primary and secondary abortion for subgroup analysis, and there was no statistical difference. Besides, IVIG can also increase the expression in peripheral blood CD3+[OR = .4, 95%CI(‐2.47, 3.15, *P* = .81],CD4+[OR = 1.16, 95%CI(‐4.60, 6.93, *P* = .69], and a decrease in the expression of CD8+[OR = ‐1.78, 95%CI(‐5.30, 1.75, *P* = .32], but there is no statistical significance.

**Conclusions:**

IVIG can significantly increase the live birth rate of recurrent spontaneous abortion. However, the evidence needs further verification and the curative effect is uncertain. It is necessary to further explore the pathogenesis of recurrent abortion and the mechanism of IVIG in the treatment of recurrent spontaneous abortion. Besides, more high‐quality randomized controlled trials suitable for population, race, dosage and timing of IVIG in the treatment of recurrent abortion are needed to confirm its effectiveness, and effective systematic evaluation is also needed to evaluate its use benefit.

## 1 INTRODUCTION

Recurrent spontaneous abortion (RSA) usually refers to the failure of pregnancy with the same sexual partner for two or more consecutive times before 20 ∼ 24 weeks.^1^ The incidence of RSA in clinic is about 12–15%, but the actual abortion rate may reach 52%, which is much higher than clinical statistics.^2^ Because of the pressure of life and childbearing concept, the incidence rate of RSA is also increasing. With the increase of the number of abortions, the recurrence risk of RSA is also increasing. Recurrent spontaneous abortion can not only cause physiological harm to patients, but also affect the psychological of patients, increasing the financial burden of patients.^3^

The etiology of recurrent abortion is very complex. At present, it is mainly considered to be related to infection, heredity, abnormal anatomical structure, mental psychology and other factors, but it has not been unified at present.^4^, ^5^ Immune factors are also important reasons.^6^ With the deepening of reproductive immunology research, people have a new understanding of many difficult problems such as pregnancy immunity, immune infertility and RSA, and gradually determine the important role of immune factors in the reproductive process. More than 60% of RSA are caused by immune system disorders, and about 80% of unexplained abortions are closely related to immune factors.^7^

Intravenous immunoglobulin belongs to passive immunotherapy. Its mechanism for the treatment of RSA is mainly to regulate cytokines secreted by lymphocytes, block the formation of complement complex, maintain maternal and fetal immune tolerance, and block maternal immune response and cytotoxicity by using antibodies in immunoglobulin.^8^, ^9^ Although a number of controlled clinical trials have been conducted to study the efficacy of IVIG in patients with RSA, there is no systematic review on RCTs of IVIG. Consequently, we performed a systematic review and meta‐analysis aiming to examine the efficacy and safety of IVIG in RSA.

## 2 METHODS

This study was conducted in accordance with Preferred Reporting Items for Systematic Reviews and Meta‐Analyses (PRISMA) .^10^ The protocol of this study was registered with PROSPERO.

### 2.1 Search strategy

We conducted an electronic search in the following databases: PubMed, Embase, Web of Science, Cochrane library, using the subject terms as below: Abortion, Habitual Or Habitual Abortion Or Habitual Abortions Or Miscarriage, Recurrent Or Recurrent Miscarriage Or Recurrent Miscarriages Or Abortion, Recurrent Or Recurrent Abortion Or Recurrent Abortions Or Recurrent Early Pregnancy Loss) AND (Immunoglobulins, Intravenous or Antibodies, Intravenous or Intravenous Antibodies or Immune Globulin, Intravenous or Intravenous Immune Globulin or Intravenous Immunoglobulins or Intravenous IG or IV Immunoglobulin or Immunoglobulins, IV or IVIG or IV Immunoglobulin or Immunoglobulin, IV or Intravenous Immunoglobulin or Immunoglobulin, Intravenous or Flebogamma DIF or Gamunex or Globulin‐N or Globulin N or Intraglobin or Intraglobin F or Intravenous Immunoglobulins, Human or Human Intravenous Immunoglobulins or Immunoglobulins, Human Intravenous or Immune Globulin Intravenous (Human) or Immunoglobulins, Intravenous, Human or Human Intravenous Immunoglobulin or Immunoglobulin, Human Intravenous or Intravenous Immunoglobulin, Human or Gammagard or Gamimune or Gamimmune or Modified Immune Globulin (Anti‐Echovirus Antibody) or Privigen or Sandoglobulin or Venoglobulin or Venoglobulin‐I or Venoglobulin I or Venimmune or Iveegam or Alphaglobin or Endobulin or Gamimune N or Gamimmune N or Gammonativ) AND (randomized Controlled Trial OR randomized OR placebo). There are no language restrictions and the last search was conducted on August 20, 2021.

### 2.2 Selection and eligibility criteria

Two authors independently conducted the title and abstract screening and full text review of the retrieval results, and the differences were resolved by consensus or the discussion with the third independent author. Inclusion criteria: RCTs on the efficacy of IVIG in the treatment of RSA patients. Only original articles were included. Exclusion criteria were as follows: non‐human studies, non‐RCTs, systematic reviews or meta‐analyses.

### 2.3 Data extraction and outcomes

Two authors independently extracted relevant data from each study into a pre‐designed Excel spreadsheet, which included country of origin, year of publication, first author, trial design, inclusion criteria, study duration, study population, intervention and duration, participant gender and age, baseline patient information and treatment outcomes. The outcomes included were live birth and T lymphocyte subsets and B cells (CD3+, CD4+, CD8+). For continuous variables, we extracted the mean and standard deviation (SD). In the absence of means and SD, the data were transformed according to the existing formulae. Differences are resolved independently by the third author.

### 2.4 Statistical analysis and quality assessment

StataMP 16 was used for statistical analysis. For continuous variables, we used standardized mean difference (SMD) and 95% CI for analysis. The I^2^ statistic was used to evaluate heterogeneity. I^2^ values of 25%, 50% and 75% were considered low, medium and high heterogeneity, respectively. Random effects models were used to pool measures in all studies. *P* values less than .05 were considered statistically significant. Risk of publication bias for studies will be assessed using Funnel plot. Quality of the RCT was assessed using the Cochrane Risk of Bias Assessment Tool, including the following six criteria: random sequence generation, allocation concealment, blinding of patients, trialists, blinding of outcome assessors, incomplete outcome data, selective reporting and other biases. Each item was assessed for risk of bias as ‘low risk’, ‘high risk’ or ‘unclear risk’ according to the recommendations of the Cochrane Handbook.

## 3 RESULTS

### 3.1 Search results

The literature search identified 1813 studies, of which 257 studies were from PubMed, 75 were from the Cochrane Library, 868 were from Embase, 611 from the Web of Science. After excluding 560 duplicates, reviewing 1253 titles and abstracts, 1171 outcomes were excluded, and the remaining 82 outcomes were reviewed in full, resulting in a total of 15 randomized controlled studies of engramine for recurrent miscarriage included in the meta‐analysis.^11^, ^12^, ^13^, ^14^, ^15^, ^16^, ^17^, ^18^, ^19^, ^20^, ^21^, ^22^, ^23^, ^24^, ^25^ The study selection process is summarized in the PRISMA flow chart (Supplementary 1).

### 3.2 Study characteristics and quality assessment

The attributes of the studies are detailed in Table S1. Four studies were conducted in 1964–2021, all 15 studies with a total of 902 more participants (trial group 479 participants used IVIG, control group 423 participants used placebo), all patients were diagnosed with recurrent miscarriage. All trials were parallel group studies with high quality according to the Cochrane Risk of Bias tool. Quality assessment results of the included studies are summarized in Supplementary 2

### 3.3 The effect of IVIG on RSA

#### 3.3.1 Live birth rate

**FIGURE 1:**
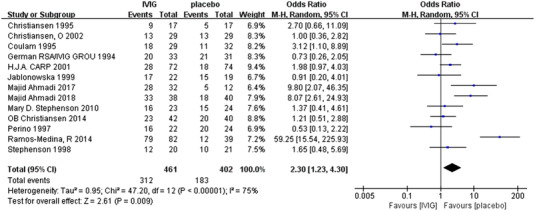
Meta‐analyses of live birth rates among categories of recurrent miscarriage

A total of 13 RCTs, reported, in a total of 863 patients, 461 IVIG users and 402 non‐users, that IVIG had the potential to improve the live birth rate of RAS patients compared to controls, the difference was statistically significant (OR 2.30[95% CI: 1.23, 4.30], *P* = .009). There was some significant difference in heterogeneity between the included studies, (*P* < .00001, I^2^ = 75%) (Figure 1).

Subgroup analysis was carried out according to whether the patients were primary or secondary recurrent abortion.

**FIGURE 2:**
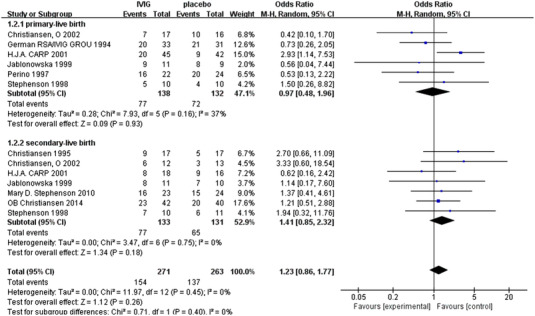
Meta‐analyses of the effect of IVIG on primary‐RSA and secondary‐RSA

Six studies reported the live birth rate of primary abortion in 270 patients, including 138 in the IVIG group and 132 in the placebo control group. There was statistical heterogeneity among the studies(*P* = .17, I^2^ = 37%). The random effect model was used for combined analysis, which shows that there was no significant difference in productivity between IVIG group and control group [OR = 1.07, 95%CI(.64, 1.78, *P* = .79]. It is suggested that the therapeutic effect of intravenous IVIG is consistent with that of placebo (Figure 2A).

A total of seven studies reported the live birth rate of secondary abortion in 264 patients, including 133 in the IVIG group and 131 in the placebo control group. There was no statistical heterogeneity among the studies (*P* = .17, I^2^ = 0%). The random effect model was used for combined analysis, which shows that there was no significant difference in productivity between IVIG group and control group[OR = 1.41, 95%CI(.86, 2.31, *P* = .17]. It is suggested that the therapeutic effect of intravenous IVIG is consistent with that of placebo (Figure 2B).

### 3.4 T cells

#### 3.4.1 CD3+

**FIGURE 3:**
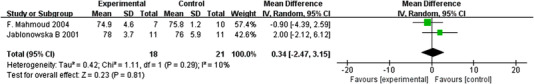
Meta‐analyses of the effect of IVIG on CD3

Two studies reported CD3 +, including 39 patients, including 18 in IVIG experimental group and 21 in placebo control group. There was statistical heterogeneity among the studies (*P* = .29, I^2^ = 10%). The random effect model was used for combined analysis, which shows that there was no significant difference in CD3 + level between IVIG group and control group[OR = .4, 95%CI(‐2.47, 3.15, *P* = .81]. It is suggested that intravenous IVIG has a tendency to reduce the expression of CD3 +, but there is no statistical significance (Figure 3).

#### 3.4.2 CD4+

**FIGURE 4:**
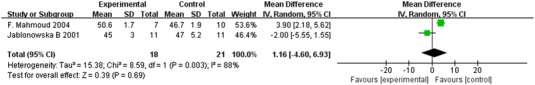
Meta‐analyses of the effect of IVIG on CD4

Two studies reported CD4 +, including 39 patients, including 18 in IVIG experimental group and 21 in placebo control group. There was statistical heterogeneity among the studies (*P* = .003, I^2^ = 88%). The random effect model was used for combined analysis, which shows that there was no significant difference in CD4 + expression between IVIG group and control group (Figure 4).

#### 3.4.3 CD8

**FIGURE 5:**
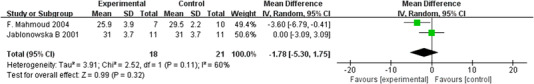
Meta‐analyses of the effect of IVIG on CD8

Two studies reported CD48, including 39 patients, including 18 in IVIG experimental group and 21 in placebo control group. There was statistical heterogeneity among the studies (*P* = .11, I^2^ = 60%). The random effect model was used for combined analysis, which shows that there was no significant difference in CD8 + expression between IVIG group and control group[OR = ‐1.78, 95%CI(‐5.30, 1.75, *P* = .32]. It suggested that the expression of CD8 + decreased after intravenous IVIG, but there was no statistical significance (Figure 5).

## 4 DISCUSSION

IVIG has high safety, few serious side effects, and no obvious side effects on the mother and offspring. The common adverse reactions are headache, fever, chills, dizziness, nausea, vomiting, muscle pain, etc., but it does not increase the risk of preterm birth. It mostly occurs in the early stage of medication and can be relieved after slowing down the infusion speed.^26^ However, the efficacy is also controversial, so our meta‐analysis of 15 randomized controlled trials aims to evaluate the effectiveness of IVIG in the treatment of RSA patients. This meta‐analysis comprehensively evaluated the effects of RS patients on live birth rate and T lymphocytes and B cells. Our results show that IVIG plays a significant role in improving the live yield. However, there was no statistical difference in the effects on T lymphocytes and B cells.

There was no significant difference in some common outcome indicators such as average birth weight and average gestational weeks between IVIG group and placebo group (*P* > .05). Therefore, this study focuses on the main outcome index ‘live birth rate’. Recurrent spontaneous abortion is divided into primary and secondary. Through meta‐analysis, it is found that IVIG treatment of recurrent spontaneous abortion has significant benefits to improve the live birth rate. However, if the study of recurrent spontaneous abortion is divided into primary and secondary, the subgroup analysis does not find a significant impact on the improvement of the live birth rate of recurrent spontaneous abortion, which may be related to the number of samples. This meta‐analysis indicated the effectiveness of IVIG on RSA. In our subgroup analysis, meta‐analysis suggested no significant difference between IVIG and placebo, but we believed that IVIG was effective on both primary and secondary RSA, even though this might be different from the existing research results.^27^ At present, the etiology in 50% of RSA patients remains unclear, and such RSA is defined as RSA with unknown cause. However, careful examination of these RSA cases with unknown cause reveals the potential immune changes, which are mainly related to the failure of fetal‐maternal immune tolerance.^28^ RSA can be classified as autoimmune type and alloimmune type, among which, alloimmune abortion is diagnosed by exclusion, namely, excluding the factors such as anatomy, chromosome, infection, endocrine and autoimmunity. Normal pregnancy belongs to the successful allogeneic hemi‐allogeneic transplantation.^29^ When analyzing from the immunology perspective, the recognition of parental antigen by the maternal immune system is a special type of peripheral immune tolerance, and it is a kind of immune protection and immune nutrition, rather than immune attack. As for embryos, blocking antibody is a protective antibody, which can not only bind to maternal lymphocyte surface antigen, but also bind to embryonic trophoblast cells, thus suppressing the immune rejection response of the mother to the fetus. Thus, the balanced regulation between immune activation and suppression at the maternal‐fetal interface is of crucial importance to maintain the normal pregnancy. Therefore, it is speculated that the occurrence of pathological pregnancies like URSA is related to the insufficient maternal blocking antibody‐induced immune attack of the fetus. Currently, immune therapy is the main treatment for antibody insufficiency. Of them, IVIG belongs to the passive immunity, which directly infuses immunoglobulins into the mother to acquire immunity, thus maintaining normal pregnancy. Establishing appropriate immune response during the implantation period is of crucial importance to the successful pregnancy, since immune factor has been verified to play an important role in pregnancy failure.^30^ The association of immune abnormality and RSA with unknown cause has been observed, which promotes the development and application of diverse immunotherapies to recover the normal immune homeostasis at the maternal‐fetal interface. Of them, one of the immunomodulator is IVIG. IVIG is the preferred treatment for patients with immunological diseases like antibody deficiency.^31^ Research suggests that the application of IVIG in treating abnormal alloimmune RSA can suppress Th1 cytokine and promote Th2 cytokine release, which is good for the balance of Th1/Th2 cytokines and thus for the maintenance of pregnancy.^32^ Additionally, some research indicates that IVIG can promote Treg cell proliferation, suppress Th17 cell proliferation and maturation, then reduce the Th17/Treg ratio, regulate its balance, enhance immune tolerance and facilitate successful pregnancy.^33^ Therefore, we conducted subgroup analysis on this basis, which revealed no significant difference compared with placebo group.

Difference in therapeutic schedule among different studies may also induce difference in results, such as the administration time of IVIG, which may affect the results, either before pregnancy or after implantation. The two RCTs regarding lymphocytes subsets enrolled in this study initiated IVIG treatment after pregnancy.^19^, ^21^ One of them administered IVIG at 500 mg/kg after the confirming of pregnancy for 5 days a month, until week 34, which is the commonly used IVIG therapeutic schedule. The other one administered IVIG after pregnancy (the dose and timing remained unclear), and both of these studies analyzed the long‐term influence of IVIG. Some studies report that the number and toxicity of NK cells at 7 days after IVIG injection decrease, and suggest that IVIG treatment should be initiated before pregnancy.^34^ Graphou et al. also pointed out that the initiation of IVIG treatment in RSA patients at the beginning of ovulatory period reduced Th1 cell number, increased Th2 cell number and declined the Th1/Th2 ratio. On the other hand, all the enrolled studies lasted till the second and third trimesters of pregnancy, consistent with the therapeutic schedule of most RSA patients. The fundamental principle of this treatment time was based on the abortion rate of 25% in RSA patients with abnormal immunity in the second trimester of pregnancy.^35^, ^36^ However, research on the short‐term effect of IVIG on lymphocyte subsets in RSA patients is lacking. Although the precise mechanism of IVIG treatment remains unclear, the lymphocyte reactivity and cytokine regulation are the cores of IVIG immune response. The therapeutic dose of IVIG remains controversial, and both high and low doses are effective.^37^ Although some studies report that the IVIG therapeutic effect is dose‐dependent,^38^ and that high dose IVIG treatment enhances the expression of CD94 suppressive receptor, which serves as another mechanism of suppressing the NK cell cytotoxicity.^39^ However, the IVIG immunoregulation seems to be qualitative, rather than quantitative. Consequently, low dose IVIG can effectively regulate the ‘Th1 to Th2 switch’ needed in successful pregnancy. Given the half‐life period of IVIG of about 23 days, treatments at intervals of 4 weeks can sufficiently regulate immunity.^40^ These remind us that when conducting IVIG RCTs, it is necessary to consider and design from multiple aspects. Besides, the different reports of these interventional strategies should be analyzed, so as to comprehensively and accurately evaluate the lymphocyte subsets and to determine the best administration timing.

T lymphocytes are derived from lymphoid stem cells in bone marrow and mature in thymus. T cells are divided into different subsets according to different CD markers on their surface. CD3 + cells are expressed on the surface of all T cells and are common surface markers of T cells. In previous studies, it was considered that there was no relationship between the expression level of CD3 + and recurrent abortion.^41^ However, recent studies have shown that the percentage of CD3 + in peripheral blood is more than 67.84%, which is conducive to pregnancy.^42^ In this study, the expression level of CD3 + increased, but there was no statistical significance. Mature cells generally only express CD4 or CD8 molecules, that is, CD4 + cells or CD8 + cells.^43^, ^44^ After the activation of CD4 + cells, the differentiated effector cells are mainly Th cells. The ratio of CD4 + cells increases, the cellular immune function increases, the rejection of embryos increases, and pregnancy cannot continue. After CD8 + cells are activated, the differentiated effector cells are cytotoxic T cells and inhibitory T cells, which can specifically kill target cells, play a negative role in immune regulation, inhibit humoral immunity, ensure that embryos are not excluded and maintain pregnancy.^45^ A large number of clinical studies show that CD8 + cells in patients with RSA are significantly reduced, and CD4 + cells and CD4 + / CD8 + ratio is significantly increased.^46^ Our study showed that CD4 + increased and CD8 + decreased after IVIG injection, but they were not statistically significant. In the hyperplastic endometrium, T cells account for about 45% of white blood cells (WBCs), even though the absolute number of T cells during the entire menstrual cycle and early pregnancy remains unchanged, but their relative number decreases with the increase in NK cell proportion.^47^ Although endometrial NK cells are different from circulatory NK cells in terms of phenotype, it is speculated that peripheral blood NK cells are closely related to NK cells in decidua.^48^, ^49^ Due to the limitation in evaluating decidual NK cells during the pregnancy, and the ability of circulating NK cells in reflecting the uterine immune status, peripheral NK cell count can be used to predict the pregnancy outcome of RSA women.^50^ Research suggests that the NK cell number in RSA patients significantly increases, after IVIG treatment, the number of NK cells decreases.^51^, ^52^ Meanwhile, some research suggests that IVIG remarkably suppresses cytotoxicity in RSA patients.^23^

Intravenous immunoglobulin is a research hotspot. At present, there are few meta‐analyses on IVIG in the treatment of RSA. This is the first meta‐analysis to comprehensively evaluate IVIG in the treatment of RSA from the aspects of live birth rate, T lymphocytes and B cells. This meta‐analysis observed heterogeneity among the enrolled studies, which might be related to the difference in study design, particularly the selection of participants. There was a great difference in the patient population, the number and age of previous spontaneous abortion were different among the patient populations. For instance, the Christiansen, O study enrolled patients experiencing at least four abortions,^18^ while some study suggested that the German RSA/IVIG Group experiment only included 19% of patients with at least four abortions, while the Stephenson MD experiment enrolled 53% of patients with at least four abortions.^11^ In addition, in some experiments, secondary RSA patients were totally excluded,^53^ while in some experiments, 50% or even 100% of secondary RSA patients were enrolled.^15^, ^16^, ^18^ Such heterogeneity may affect the experimental results, since many studies suggest that, secondary abortion women with a history of at least four to five or more abortions are more prone to develop the immune background than those with less abortions.^54^, ^55^ On the other hand, age is also an important aspect of population difference. The application of IVIG in the treatment of RSA remains controversial. In some of our enrolled studies, the significant benefits of IVIG were demonstrated, but in RCTs not proving such benefits, the absence of benefits was ascribed to the improper selection of patients, so the elderly women were rejected.^11^, ^12^, ^56^ Some research suggests that, compared with young women with a history of recurrent abortions, the elderly women with recurrent abortions are more likely to have immune problems.^53^, ^57^ Other problems including the insufficient screening of patient immune abnormality have not been solved in large multi‐center trials.^40^, ^53^, ^57^ It was concluded in this study that, although IVIG treatment was not beneficial for primary RSA patients, its overall therapeutic effect on RSA patients or secondary RSA patients remained to be further determined. Further high‐quality studies require an enough number of participants and rigid research design. The future studies should focus on solving the following problems that may affect results. The studies should adopt the double‐blinding and randomization methods to reduce bias. The studies should control the participant age, history of spontaneous abortion, endocrine status, genetic polymorphism and immunological characteristics; determine the optimal timing, the most effective and optimal doses of IVIG.

Overall, our results show that IVIG can significantly increase the live birth rate of recurrent spontaneous abortion, but this study has some limitations and uncertain curative effect. To conclude, it still has a good research prospect in the treatment of recurrent abortion caused by immune abnormalities. It is necessary to further explore the pathogenesis of recurrent abortion and the mechanism of VIG in the treatment of recurrent spontaneous abortion. It also needs more high‐quality randomized controlled trials suitable for the population, race, dosage and timing of IVIG in the treatment of recurrent abortion to confirm its effectiveness, as well as effective systematic evaluation to evaluate its use benefit.

## Supporting information

Supporting Information

Supporting Information

Supporting Information

Supporting Information

Supporting Information

## Data Availability

Data sharing not applicable to this article as no datasets were generated or analysed during the current study.
