# Supplementary material for: Efficacy of intravenous immunoglobulin in the treatment of recurrent spontaneous abortion: A systematic review and meta‐analysis
Source: Am J Reprod Immunol. 2022 Aug 30;88(5):e13615. doi: 10.1111/aji.13615 (PMC9787751; doi:10.1111/aji.13615)
Supplement: Supplementary file 1 — Supporting Information [file AJI-88-e13615-s002.docx]

1. PRISMA flow chart


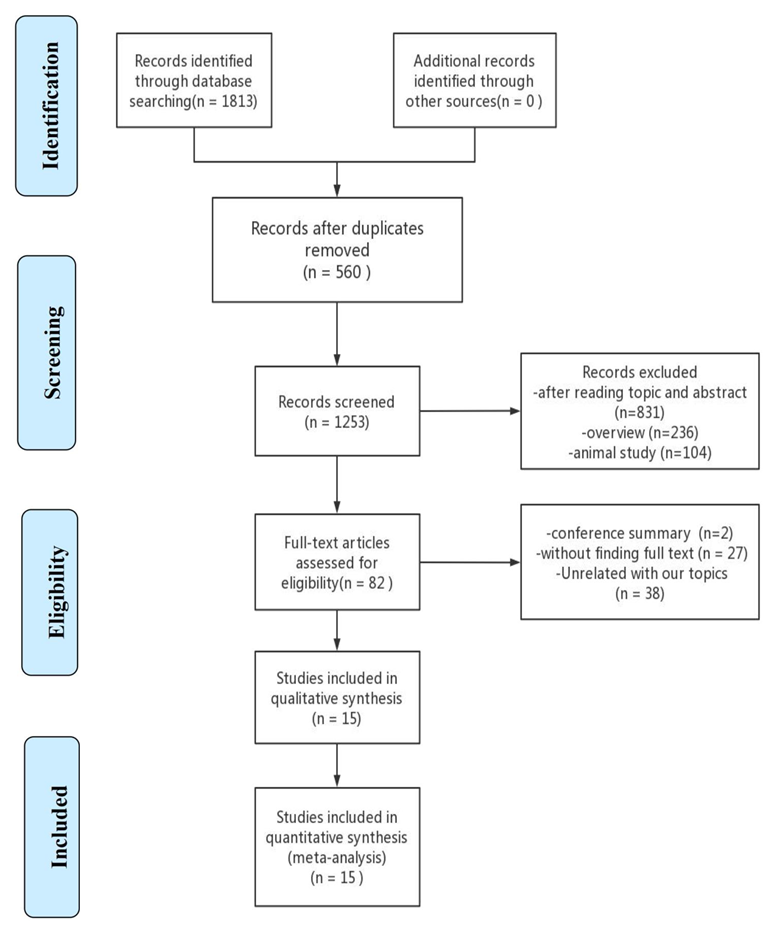


1. Quality assessment of included studies

**
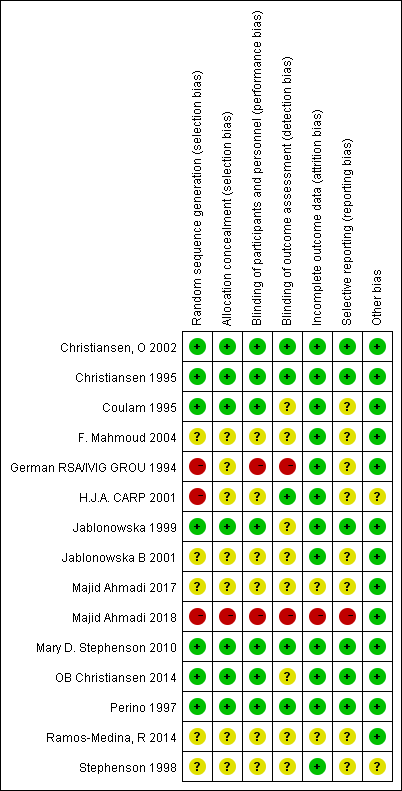
**

**
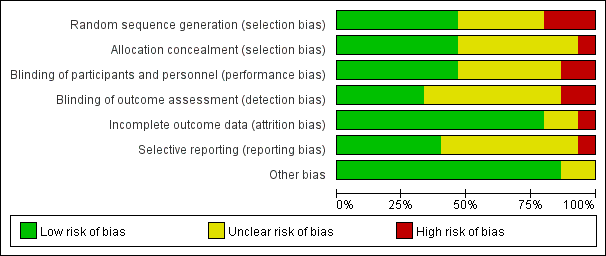
**

| **Included studies** | **DATE** | **Treatment group** | | | **Control group** | | |
| --- | --- | --- | --- | --- | --- | --- | --- |
|  |  | **Intervention** | **Number of live births** | **Number of achieved pregnancies** | **Intervention** | **Number of live births** | **Number of achieved pregnancies** |
| Christiansen | 1995 | IVIG | 9 | 17 | Human albumin | 5 | 17 |
| Coulam | 1995 | IVIG | 18 | 29 | 5% albumin | 11 | 32 |
| Perino | 1997 | IVIG | 16 | 22 | 5% albumin | 20 | 24 |
| Stephenson | 1998 | IVIG | 12 | 20 | Normal saline | 10 | 21 |
| Jablonowska | 1999 | IVIG | 17 | 22 | Normal saline | 15 | 19 |
| H.J.A. CARP | 2001 | IVIG | 28 | 72 | Human albumin | 18 | 74 |
| Christiansen, O | 2002 | IVIG | 13 | 29 | Human albumin | 13 | 29 |
| OB Christiansen | 2014 | IVIG | 23 | 42 | Normal saline | 20 | 40 |
| Majid Ahmadi | 2017 | IVIG | 28 | 32 | Not depicted | 5 | 12 |
| Majid Ahmadi | 2018 | IVIG | 33 | 38 | Not depicted | 18 | 40 |
| Ramos-Medina, R | 2014 | IVIG | 79 | 82 | Not depicted | 12 | 39 |
| Mary D. Stephenson | 2010 | IVIG | 16 | 23 | Normal saline | 15 | 24 |

1. Randomised controlled trials pertaining to recurrent miscarriage
