# Supplementary figures and images for: Efficacy of intravenous immunoglobulin in the treatment of recurrent spontaneous abortion: A systematic review and meta‐analysis
Source: Am J Reprod Immunol. 2022 Aug 30;88(5):e13615. doi: 10.1111/aji.13615 (PMC9787751; doi:10.1111/aji.13615)

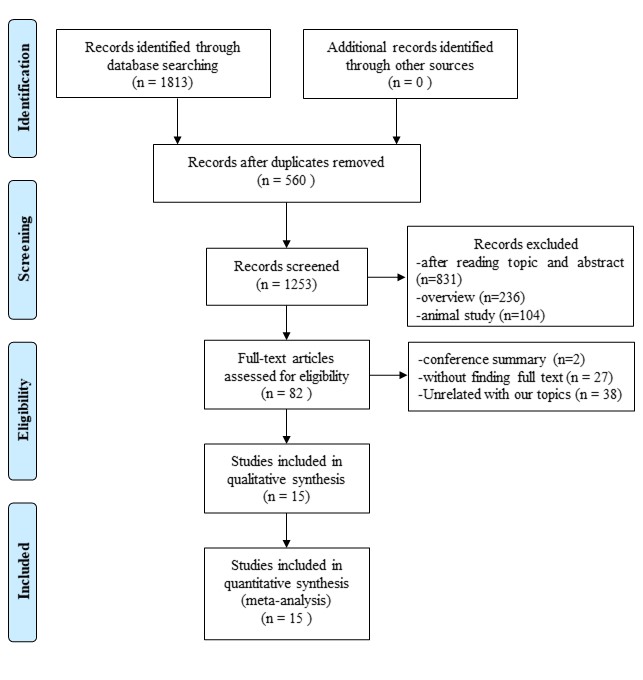

Supplement: Supplementary file 2 — Supporting Information [file AJI-88-e13615-s004.jpg]

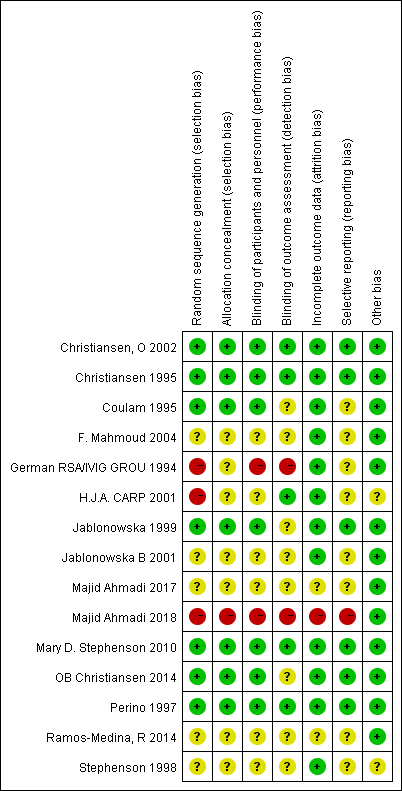

Supplement: Supplementary file 3 — Supporting Information [file AJI-88-e13615-s003.png]

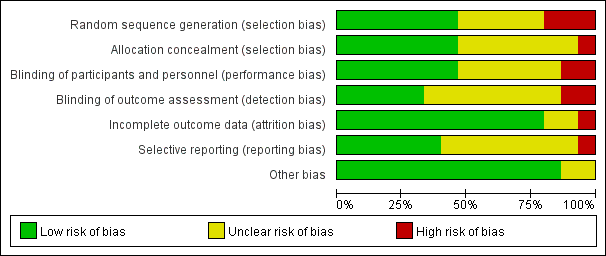

Supplement: Supplementary file 4 — Supporting Information [file AJI-88-e13615-s001.png]
